# An evaluation of the tumour endothelial marker CLEC14A as a therapeutic target in solid tumours

**DOI:** 10.1002/cjp2.176

**Published:** 2020-07-21

**Authors:** Joseph Robinson, Katharine Whitworth, Elizabeth Jinks, Zsuzsanna Nagy, Roy Bicknell, Steven P Lee

**Affiliations:** ^1^ Institute of Immunology and Immunotherapy University of Birmingham Birmingham UK; ^2^ Institute of Inflammation and Ageing University of Birmingham Birmingham UK; ^3^ Institute of Cardiovascular Sciences University of Birmingham Birmingham UK

**Keywords:** CLEC14A, tumour endothelial marker, immunohistochemistry, renal cell carcinoma, cancer, chimeric antigen receptor

## Abstract

Earlier studies identified the transmembrane cell surface C‐type lectin CLEC14A as a putative tumour endothelial marker. For CLEC14A to progress as a vascular target in solid tumours an in‐depth analysis of CLEC14A expression in human healthy and tumour tissue is needed. It is here shown that an analysis of 5332 RNA expression profiles in the public domain confirmed high expression of *CLEC14A* in tumour compared to healthy human tissue. It is further shown by immunohistochemistry that CLEC14A protein is absent, or expressed at a very low level, in healthy human and primate tissue. In contrast, CLEC14A is expressed on the vasculature of a range of human solid tumours, with particularly high expression in more than half of renal cell carcinomas. Elevated levels of *CLEC14A* transcripts were identified in some non‐cancer pathologies; such comorbidities may need to be excluded from trials of therapies targeting this marker. It is further shown that, as CLEC14A expression can be induced by the absence of shear stress, it is imperative that freshly collected as opposed to aged or post‐mortem tissue be analysed. We conclude that CLEC14A is a promising target to enable development of novel anti‐cancer therapies for solid tumours.

## Introduction

C‐type lectin domain family 14 member A (CLEC14A) is a protein expressed on the surface of endothelial cells. It is known to play a role in angiogenesis, mediating cell–cell contacts, endothelial cell migration and tube formation. In healthy tissues, expression of CLEC14A is regulated by shear stress; thus, endothelial cells (human umbilical vein) grown under static conditions express high levels of CLEC14A, but expression is greatly reduced under physiological flow [1]. Earlier studies of CLEC14A expression reported that it is upregulated in many solid tumours including those of the ovary, prostate, breast, liver, bladder, kidney and lung [1, 2]. This may partly reflect the reduced flow rate often present within tumour vasculature [3]; however, recent studies indicate that, in cancer, CLEC14A expression may be regulated by other pathways [4]. Poor tumour growth in CLEC14A (−/−) mice compared to wild type mice confirmed that CLEC14A promotes tumour growth [5].

High expression of CLEC14A on tumour blood vessels and its apparent role in tumour vascularisation make it an attractive target for tumour vasculature‐targeted immunotherapy. Indeed, antibody blockade of CLEC14A interactions with its natural ligand, the endothelial‐specific extracellular matrix protein multimerin‐2, reduces angiogenesis and tumour growth in a mouse model [5]. Furthermore, recent clinical studies have demonstrated the remarkable potency of cytotoxic T‐cell‐based therapies targeting cancer antigens, and with this in mind we have developed T‐cells engineered with chimeric antigen receptors (CARs) that target CLEC14A on the tumour vasculature. These CAR T‐cells specifically lyse CLEC14A‐expressing cells *in vitro* and reduce tumour growth in several mouse models (manuscript submitted).

Clearly, the clinical efficacy of such therapeutic approaches is dependent on the expression pattern of CLEC14A. Given the potency of T‐cell‐based therapies, expression of the target antigen on healthy tissues presents a risk of serious side effects, as has been reported with other putative tumour antigens [6, 7, 8, 9, 10]. Thus, to identify tumour antigens that may be safely targeted there is a critical need to characterise their expression in healthy tissues. In this way we can predict potential on‐target off‐tumour toxicities to guide the design of safety studies, select patients likely to benefit from treatment and exclude at‐risk patients.

Here we report a comprehensive analysis of CLEC14A gene and protein expression in healthy, cancer and other diseased human tissues and also in informative healthy primate tissues.

## Methods

### Analysis of public databases for *CLEC14A* transcript expression in healthy and cancer tissues

To further validate the tumour‐specific expression of *CLEC14A*, data available from public datasets were analysed. Initially the expression pattern of *CLEC14A*, several endothelial markers (*PECAM1*, *vWF* and *TIE1*) and β‐actin (*ACTB*) in a collection of healthy tissues (GeneAtlas U133A, gcrma, http://biogps.org) was studied. Expression data were logged and normalised to *ACTB*. Then we accessed the GEO database (Gene Expression Omnibus, http://www.ncbi.nlm.nih.gov/geo/) collecting maximum data on the same genes in healthy tissue, cancer and non‐cancer pathologies. Data were obtained on 5332 samples from 133 studies, with samples from a wide representation of organs (see supplementary material, Tables S1 and S2). For further details see Supplementary materials and methods.

TIE1 expression in endothelial cells is a validated marker of reduced sheer stress [11, 12, 13] so we used the ratio of *TIE1*/*vWF* or *Tie1*/*PECAM1* expression to measure flow rates whilst correcting for differences in vascular density between samples. The ratio of *CLEC14A*/*PECAM1* was taken as the expression of *CLEC14A* per endothelial cell correcting for differences in vascular density between samples (with the assumption that *PECAM1* expression is the same in healthy and tumour endothelial cells). The ratio of *CLEC14A*/*TIE1* was taken as a measure of *CLEC14A* expression normalised for variations in blood flow in the tissues.

To allow group comparisons, all ratios were standardised using *z* scores. *Z* scores were calculated using the mean and standard deviation of each measure derived from the healthy tissue reference dataset (GeneAtlas U133A). Using the ratio of *TIE1*/*vWF* we could further subdivide samples from the GEO dataset based on this measure of blood flow (*TIE1*/*vWF* ratio < 0.7 was regarded as an indicator of healthy flow based on an analysis of healthy tissues (see supplementary material, Figure S1A and Table S3).

### Tissue samples

Fresh tonsil samples and frozen human placenta were obtained from the Human Biomaterials Resource Centre at the University of Birmingham with ethical approval (reference 13‐153). Human tissue microarrays (TMAs) used in this study were purchased from Pantomics (Richmond, CA, USA; arrays MNO961, MNO1021, MTU481, KIC1021, LVD481, BRC481 and LUD481), where surgical samples were fixed within 30 min of removal by immersion in 10% neutral buffered formalin for 24 h. Note that autopsy samples were fixed up to 8 h post‐mortem. Cynomolgus macaque tissues, frozen within 1 h of cessation of circulation, were purchased from the Biomedical Primate Research Centre (Rijswijk, The Netherlands).

### Immunohistochemistry: Human tissue

Tissue sections were stained for CD31 with antibody JC70A (Dako, Santa Clara, CA, USA), followed by ImmPRESS Reagent Peroxidase Anti‐Mouse. For staining of CLEC14A, TMAs were incubated with a polyclonal primary antibody AF4968 (0.85 μg/ml; R&D Systems, Minneapolis, USA) or concentration matched isotype control (5‐001‐A; R&D System), followed by anti‐sheep HRP (R&D System). For further details see Supplementary materials and methods.

Immunohistochemical staining was assessed by the authors then independently validated (independent staining through to analysis) by a specialist contract research organisation (Propath, UK). CLEC14A staining intensity was scored on a scale of 0–4, 0 for no staining and 4 for intense staining. The percentage of CLEC14A positive vessels was determined by comparing the number of CLEC14A stained vessels to the number of CD31 stained vessels in a serial section. The Colour Thresholding feature of ImageJ software (Version 1.48; NIH) was used to determine the vascular coverage of samples as a percentage of tissue area. Staining scores were determined by multiplying together the intensity score, percentage CLEC14A‐positive vessels and percentage vascular coverage. Samples with non‐specific staining for either CLEC14A or CD31 were excluded from the analysis. Receiver operating characteristic (ROC) analysis of staining scores was performed using MedCalc for Windows (MedCalc Software, Ostend, Belgium).

### Immunohistochemistry: Cynomolgus macaque tissue

CLEC14A expression in frozen Cynomolgus macaque tissue (taken from 3 male animals, aged 8, 8 and 12 years) was determined using an anti‐CLEC14A mouse monoclonal antibody (CRT3) that was generated according to the method described by Noy *et al* [5]. Staining of CLEC14A and CD31 was carried out using the ImmPRESS Excel Amplified HRP Polymer Staining Anti‐mouse IgG kit (Vector Laboratories, Burlingame, CA, USA), as per manufacturer instructions (see Supplementary materials and methods).

## Results

### Analysis of public databases for *CLEC14A* transcript expression in healthy and cancer tissues

To evaluate the potential of CLEC14A as a target for safely treating solid tumours, we explored publicly available datasets to compare gene expression in healthy, cancer and other diseased human tissues. However, given that CLEC14A is known to be an endothelial marker and its expression is affected by altered blood flow rates [1], we first needed to determine the best way in which to analyse these datasets to correct for differences in vascular density between tissues and possible delays in tissue processing after cessation of blood flow. Therefore, we began by analysing a single dataset from healthy tissues for expression of different endothelial markers and *CLEC14A*.

### Expression of endothelial marker genes (*PECAM* and *vWF*) in healthy tissues

Analysing the GeneAtlas U133A dataset of healthy tissues, we found levels of endothelial markers (as a proxy for vascular density) to vary across tissues, with *PECAM1* (ranking scores = 17.88–53.86) and *vWF* (ranking scores = 26.84–82.44) showing the highest expression in the lung, heart, uterus and placenta (amongst others) (see supplementary material, Figure S1A). The expression of these two endothelial markers in healthy tissues showed a strong correlation (*p* < 0.0001, *R* ^2^ = 35.11%). The expression pattern of these markers in the different tissues and their relationship is to be expected from the literature [14]. Using the ratio of *TIE1*/*vWF* expression as a marker of shear stress, in healthy tissues this ratio ranged between 0.10 and 0.69 (mean: 0.43), with the higher ratios found in lung, liver, heart and placenta. This is in agreement with the lower or more disturbed shear stress in these tissues [15] (see supplementary material, Figure S1B).

### Expression of the *CLEC14A* gene in healthy tissues

**Figure 1:**
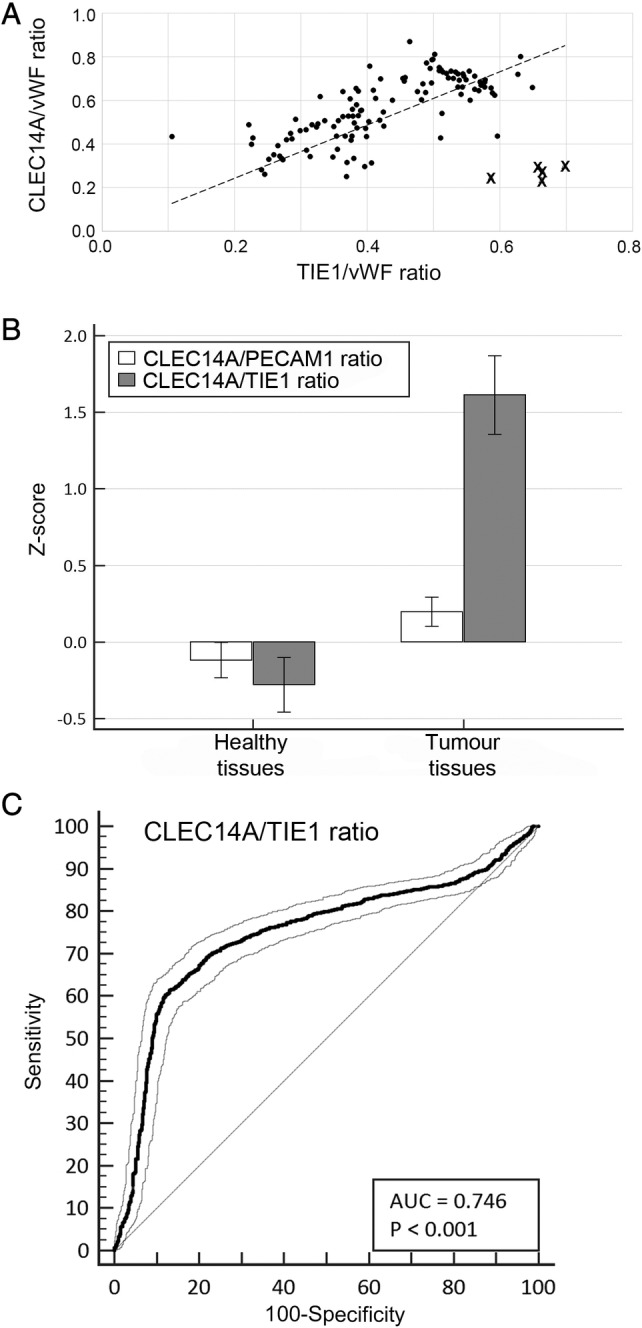
Gene expression analysis of GEO datasets. (A) Regression analysis of *CLEC14A*/*vWF* and *TIE1*/*vWF* ratio in healthy tissues. Crosses indicate the five outliers. Dashed line: linear regression line: *p* < 0.0001, *R* ^2^ = 53.74%. (B) Comparison of *CLEC14A*/*PECAM1* and *CLEC14A*/*TIE1* expression ratios in healthy and tumour samples. Data shown represent the mean *z*‐score including standard deviation of the mean. Tumour samples include only untreated primary and metastatic tumours. The control group includes only tissue samples obtained from healthy individuals (Healthy tissues). (C) ROC curve analysis showing the diagnostic power of the *CLEC14A*/*TIE1* ratio for untreated tumours. ROC curve with 95% CIs.

In healthy tissues, *CLEC14A* gene expression was low (ranking scores = 17.45–27.07, mean: 20.71, SD: 1.2). Of all tissues the skeletal muscle had the highest expression while the lowest expression was in the frontal cortex (see supplementary material, Figure S1A). However, there was little variation between tissues, and *CLEC14A* levels did not correlate with either of the two endothelial markers (*PECAM1* or *vWF*). This supports previous reports that CLEC14A shows very low expression in healthy tissues irrespective of the vascular density [1]. However, there was a significant positive relationship between the *TIE1*/*vWF* ratio and the *CLEC14A*/*vWF* ratio (*p* < 0.0001, *R* ^2^ = 53.74%), showing that in healthy tissues low shear stress is associated with increased *CLEC14A* expression (Figure 1A).

### Using *CLEC14A*/*PECAM* and *CLEC14A*/*TIE1* ratios for data analysis

CLEC14A is a sheer stress regulated endothelial marker. Therefore, when comparing the expression of *CLEC14A* in different tissues, the variations of vascular density and the lack of flow in tissues (due to delayed processing such as occurs with *post mortem* tissues) need to be taken into account. Consequently, we corrected for these factors using ratios of *CLEC14A*/*PECAM1* (to describe the *CLEC14A* expression per endothelial cell) and *CLEC14A*/*TIE1* (to correct for differences induced by flow).

Using the analysis of healthy tissue data from the GeneAtlas U133A dataset we established the reference intervals for the different endothelial markers and *CLEC14A* (see supplementary material, Figure S2A–F). While the expression of endothelial markers *vWF* and *PECAM* in different healthy tissues showed a wide variation (see supplementary material, Figures S2A–B), *TIE1* levels expressed as a ratio of *PECAM* or *vWF* were relatively stable amongst the healthy tissues. Similarly, there was relatively little variation in the ratio of *CLEC14A*/*PECAM* and *CLEC14A*/*TIE1* (see supplementary material, Figure S2C–F).

In the analysis of tumour tissues, two datasets from the GEO database included repeat sampling of tumour tissue. Study GDS2416 included samples from cervical cancer patients where several biopsies were taken from the same patient at single time‐points. The data indicate that even within the same tumour, tissue vascularity (as defined by *PECAM1* expression) is highly variable (see supplementary material, Figure S3A) as is *CLEC14A* expression (see supplementary material, Figure S3B). However, correcting for differences in vascular density and blood flow rates by measuring *CLEC14A*/*PECAM1* and *CLEC14A*/*TIE1* ratios respectively gave relatively consistent readings in repeat samples from the same patient. Larger variations in *CLEC14A*/*PECAM1* ratios only occurred for the same patient where the tissue vascularity in some repeat samples was very low (patients P1 and P10).

Study GDS4547 processed samples from the same renal carcinoma tumours at different temperatures and after different time periods to assess the effects of storage and tissue hypoxia on the expression of different genes. Using this dataset to analyse the effect of these variables on gene expression, we found that although some genes such as vimentin altered expression levels significantly over the 2 h (especially when held at 37 °C [16]), *CLEC14A*/*PECAM1* and *CLEC14A*/*TIE1* ratios showed no significant changes (see supplementary material, Figure S4).

Based on these two datasets, it appears that whilst the *CLEC14A*/*PECAM1* and *CLEC14A*/*TIE1* ratios can vary between patients, they are relatively stable in samples taken from the same patient (see supplementary material, Figure S3). Furthermore, there are no clear time or temperature dependent changes in these ratios that would distort results due to unreported differences in tissue processing (see supplementary material, Figure S4). Therefore, we used these more stable markers to analyse a much larger number of healthy and tumour tissues from the GEO dataset (see below) as they should allow a more reliable measure of the differences between tissues.

### Tumour specificity of *CLEC14A*: A meta‐analysis of 133 GEO datasets

Having established the most reliable way to analyse expression of *CLEC14A* in the vasculature, we conducted a meta‐analysis of 133 GEO datasets to compare expression of this marker in healthy and diseased tissues. Overall, *CLEC14A* mRNA expression per endothelial cell (expressed as *CLEC14A*/*PECAM* ratio) was significantly higher in the tumour group relative to healthy tissues (Figure 1B). The same was also true of the flow‐adjusted *CLEC14A* expression (*CLEC14A*/*TIE1* ratio). In fact, the difference in the *CLEC14A*/*TIE1* ratio alone was significant enough to allow the correct identification of sample status (healthy or tumour) in almost 75% of samples (Figure 1C).

We found that high *CLEC14A*/*PECAM1* and *CLEC14A*/*TIE1* ratios (ratio above 95% of controls) were significantly associated with tumour pathology (odds ratio = 2.5113, 95% CI: 1.7467–3.6104 and odds ratio = 8.9821; 95% CI: 6.7440–11.9630 respectively). While the overall effects were strong, the effects were tissue dependent (see supplementary material, Tables S4 and S5). *CLEC14A* expression in tumour tissues (normalised to *PECAM1* or *TIE1* expression) is also significantly above most, but not all, non‐tumour pathologies (see supplementary material, Table S6).

### CLEC14A protein expression in human tissue

**Figure 2:**
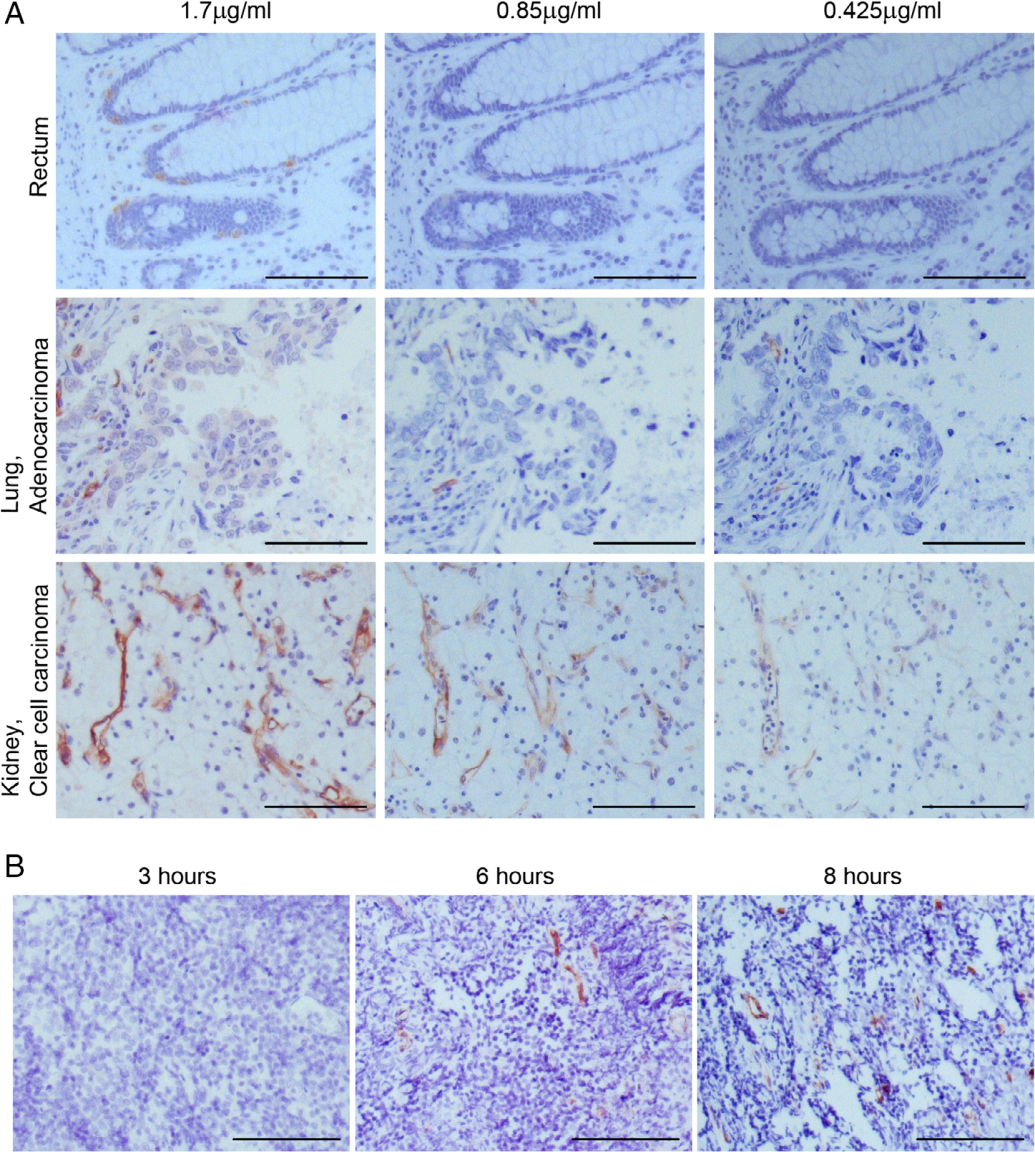
Optimisation of immunohistochemical staining of CLEC14A in human tissue. (A) Antibody titration: representative images of CLEC14A staining using polyclonal serum on formalin fixed paraffin embedded TMAs at three concentrations of CLEC14A antibody (1.7, 0.85 and 0.425 μg/ml). (B) Delayed tissue processing increases CLEC14A expression: representative images of CLEC14A staining using polyclonal serum on tonsil tissue (*n* = 2) stored for increasing lengths of time (3, 6 and 8 h) prior to formalin fixation. Scale bars = 100 μm.

Analysis of transcripts does not necessarily inform on protein expression levels. Therefore, to complement the studies on RNA expression, we performed immunohistochemical analysis of tissue sections to compare CLEC14A protein expression in healthy and diseased tissues. Initial studies explored the optimum concentration of primary antibody for CLEC14A staining of fixed human tissue. This was determined through titration of the antibody on the TMAs (Figure 2A). At the highest concentration (1.7 μg/ml) non‐specific (i.e. non‐endothelial) staining was observed in some tissues. After a two‐fold dilution (0.85 μg/ml) non‐specific staining disappeared but specific staining on tumour vasculature remained. Further dilution resulted in loss of specific staining; therefore 0.85 μg/ml was selected as the optimum staining concentration for use on the TMAs.

As CLEC14A expression is regulated by blood flow, long tissue processing times have the potential to mislead the results. To test this, fresh tonsil tissue was immediately stored at 4 °C in RPMI 1640 (Sigma, Sigma‐Aldrich, UK) for 3, 6 or 8 h prior to fixation for 24 h in 10% neutral buffered formalin and paraffin embedding. Storing tonsil tissue for 6 h before fixation was shown to cause an upregulation of CLEC14A expression (Figure 2B). Therefore, where possible, studies of CLEC14A expression were conducted using tissues processed within 30 min of surgery or death of the donor.

**Figure 3:**
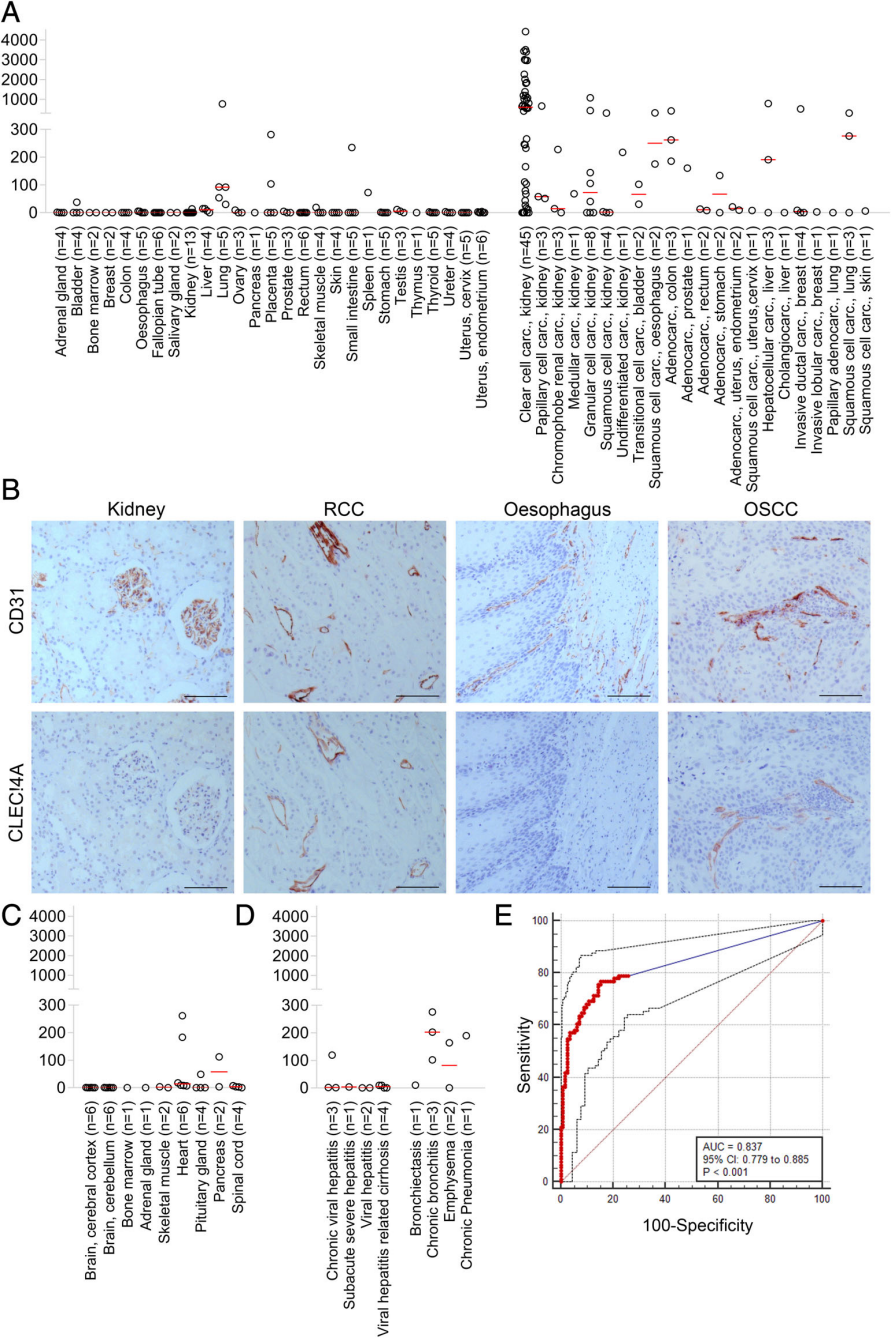
CLEC14A expression in healthy, cancer and non‐cancer pathologies. (A) CLEC14A staining scores for surgical samples from healthy (left) and tumour (right) tissue. Red line = median score. (B) Representative images of CD31 and CLEC14A staining on healthy kidney, renal clear cell carcinoma (RCC), healthy oesophagus and oesophageal squamous cell carcinoma (OSCC). Concentration matched isotype control antibody staining was negative. Scale bars = 100 μm. (C) CLEC14A staining scores for autopsy samples of normal human tissues. Red line = median score. (D) CLEC14A staining scores for surgical samples of non‐cancer diseased human tissues. Red line = median score. (E) ROC curve analysis of staining score in tumour tissues (*n* = 91) relative to control samples (*n* = 112).

Studying a wide range of rapidly processed healthy human tissues, we found CLEC14A immunostaining was confined to endothelial cells (which were identified by morphology and CD31 staining of a serial section) but in the majority of healthy tissues CLEC14A expression was weak or absent (Figure 3A,B). Weak to moderate staining was present in the alveoli of all lung samples stained. Weak to moderate staining was also present in 2 of 5 placenta samples, 1 of 5 small intestine samples and 1 of 1 spleen samples. With healthy tissues obtained from autopsy (where tissue processing times could be up to 8 h post mortem), moderate staining was observed on 2 of 6 heart samples and 1 of 2 pancreas samples (Figure 3C). Staining on other autopsy tissues, namely brain, bone marrow, adrenal gland, skeletal muscle, pituitary gland and spinal cord, was weak or absent.

Analysis of tumour tissues again used samples that had been processed within 30 min of surgery. CLEC14A expression on tumour samples was variable (Figure 3A,B). In many samples, staining was present on most or all of the vessels but in others little or no staining was observed. During the initial staining of tumour tissues, we identified expression in renal clear cell carcinoma to be particularly high so we focused our analysis on this tumour type and demonstrated strong staining (staining score > 400) in 58% of clear cell renal cell carcinoma samples.

Studying non‐cancerous diseased human tissues, weak to moderate CLEC14A staining was detected on endothelial tissues in diseases of the lung such as chronic bronchitis, emphysema and chronic pneumonia (Figure 3D), resembling that seen in healthy lung tissues (Figure 3A). In diseases of the liver, including various forms of hepatitis, CLEC14A staining was generally weak or absent.

ROC curve analysis of CLEC14A staining scores was performed in order to determine the sensitivity and specificity of CLEC14A expression levels in tumour tissues relative to healthy tissue samples. Analysis of 112 normal tissues and 91 tumour samples indicated that CLEC14A expression accurately predicts tumour status (accuracy AUC = 83.7%; 95% CI: 0.779–0.885; *p* < 0.001) with sensitivity and specificity values above 75 and 85% respectively (Figure 3E). These data support the proposal that CLEC14A as a target is selective for tumours and indicate that a biomarker assay could be developed for diagnostic (including companion diagnostic) purposes.

### CLEC14A protein expression in *Cynomolgus macaque* tissue

Certain human tissues (e.g. heart, brain and spinal cord) are only available as autopsy samples, and are likely to have experienced prolonged periods without flow prior to fixation. As described above, staining these tissues for CLEC14A may therefore lead to false positive results. Hence, as a separate study, we analysed protein expression in tissue from *Cynomolgus macaques* which had been frozen within 1 h after culling. CLEC14A from this species shares 94% amino acid sequence identity with human CLEC14A.

**Figure 4:**
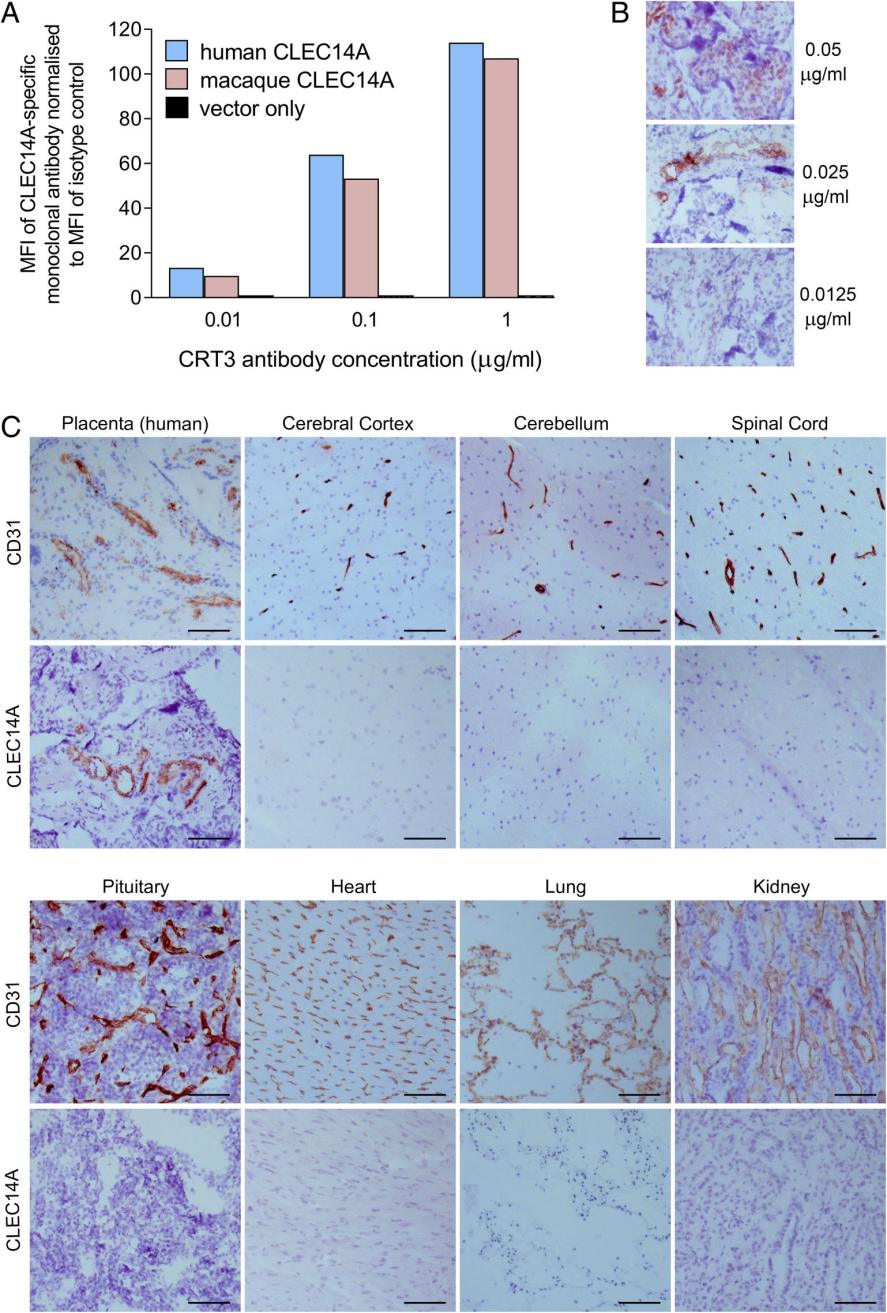
CLEC14A protein expression in cynomolgus macaque tissues. (A) Flow cytometric analysis of CRT3 staining of human and cynomolgus macaque CLEC14A‐expressing HEK293T cells. Mean fluorescence intensity (MFI) was normalised by dividing by the MFI of wild type HEK293Tcells stained at the same antibody concentration. (B) Optimisation of immunohistochemical staining for CLEC14A in frozen human placenta sections using the CRT3 monoclonal antibody. Representative images shown. (C) Images of CD31 and CLEC14A staining on frozen sections of human placenta and cynomolgus macaque tissues. Concentration‐matched isotype control antibody staining was negative. All scale bars = 100 μm.

In order to determine if the CLEC14A specific monoclonal antibody CRT3 stains human and macaque CLEC14A equally, 293T cells were transduced with lentivirus expressing the human or cynomolgus macaque *CLEC14A* genes and co‐expressing GFP. The transduced 293 T cells (identified by GFP expression) were then stained with titrating concentrations of CRT3. Flow cytometric analysis revealed similar levels of staining between the two cell lines at all concentrations of CRT3 tested (Figure 4A). In contrast, no staining above background was seen with 293T cells transduced with the empty control vector. These findings demonstrate the specificity of the CRT3 monoclonal antibody for CLEC14A, and suggest that CLEC14A staining using this antibody on human and macaque tissues is comparable.

Therefore, we then used the same CRT3 antibody to stain frozen human placenta (positive control) and frozen tissues from three healthy *Cynomolgus macaques* (*n* = 3). The optimum concentration of this antibody for CLEC14A staining was determined through titration studies using frozen human placenta sections as a positive control (Figure 4B). A concentration of 0.025 μg/ml was selected as the optimum concentration as it gave strong endothelial staining without non‐specific staining. CLEC14A expression was clearly detected in the vessels of human placental tissue, as indicated by co‐staining a serial section of this tissue for the endothelial marker CD31 (Figure 4C). In contrast, healthy cynomolgus macaque tissues including cerebral cortex, cerebellum, spinal cord, pituitary, heart, lung and kidney stained in parallel with the human placenta, showed no evidence of CLEC14A expression. These primate tissues stained strongly for CD31 indicating that endothelial cells were present within the sections.

## Discussion

Expression of CLEC14A on tumour vasculature makes it a potential target for vascular disruption agents such as potent new cytotoxic immunotherapies. However, if it is to be safely targeted it is critical to understand the expression pattern of this molecule in healthy and non‐cancerous diseased tissues. Therefore, we have undertaken an extensive analysis in both human and primate tissues.

One of the key lessons from this study is the importance of how tissues are collected and prepared for analysis. CLEC14A is known to be regulated by changes in flow rate [1] and therefore we hypothesised that different processing times before fixation of tissue could greatly affect studies on expression of this molecule. Our findings, both for RNA and protein expression, support this hypothesis and have important implications for studying the expression of any gene that is regulated by shear stress. Previous work using microarray analysis of HUVEC cells indicated that sheer stress can regulate expression of up to 350 genes [3]. In this regard, another consideration is the size of the tissue collected, since larger tissues will take longer for the fixative solution to perfuse the sample. It was ensured that all tissues in the present study were no larger than 1 cm^3^.

The meta‐analysis of expression data from the GEO database indicated that expression of *CLEC14A* transcripts in healthy tissues is generally very low and is mainly dependent on the shear stress induced by blood flow. In tumours, however, high *CLEC14A*/*TIE1* ratios indicate that *CLEC14A* mRNA levels are significantly higher in endothelial cells than could be expected based on shear stress alone. This supports the hypothesis that different mechanisms, besides turbulent flow, contribute to the expression of this marker on endothelium in some tumours. A recent report indicates that *CLEC14A* expression is directly controlled by signalling through the activin receptor‐like kinase 1 (ALK1), a type I receptor of the TGF‐beta superfamily [4]. Bone morphogenetic protein (BMP)‐9 and BMP‐10 bind the ALK1 receptor leading to signalling in endothelial cells through SMADs 1, 5 and 8 to mediate development of blood vessels. *In vitro* studies indicate that, in contrast to BMP‐9 which upregulates *CLEC14A* expression in endothelial cells, TGF‐beta downregulates *CLEC14A* expression. Thus, *CLEC14A* expression in vessels appears to be controlled by several factors and it is likely to be a combination of these that will determine which tumours and tissues show elevated levels.

Analysing *CLEC14A*/*TIE1* ratios in the GEO database indicates that, for some healthy tissues, expression of *CLEC14A* transcripts may be elevated (see supplementary material, Table S4), particularly in healthy breast tissue, and occasionally in liver or lung. This could indicate the potential for toxicity since therapies targeting CLEC14A might also attack these healthy tissues. Although studying gene expression databases permits analysis of large numbers of samples, the presence of a transcript does not necessarily confirm expression at the protein level. Therefore, in addition to analysing the GEO dataset, we conducted immunohistochemical studies on human tissue arrays. Exploring such tissues for CLEC14A protein expression we did not detect it in healthy breast (Figure 3, *n* = 2). Further studies on another 12 healthy breast tissues showed relatively few vessels, but only two tissues showed any staining for CLEC14A protein and this was at a very low level. Should breast tissue be targeted by on‐target off‐tumour toxicity this is likely to be manageable. We did not detect CLEC14A protein in healthy liver tissues, and although we did detect some CLEC14A protein in lung tissue this was at a much lower level than that seen in some tumours. Returning to our analysis of the GEO dataset, higher *CLEC14A*/*TIE1* ratios were also detected in some other healthy tissues but these were only in a small minority of cases. For example, with healthy stomach and thyroid tissues, 12–15% of cases had a high *CLEC14A*/*TIE1* ratio but this represented only 1 out of a total of 7 or 8 cases respectively (see supplementary material, Table S4). Thus, one of the limitations of our study is that, despite analysing over 5000 samples, for some healthy tissues data were only available on relatively few cases. For healthy bladder tissue only one case was available for analysis from the GEO dataset and this one case had a high *CLEC14A*/*TIE1* ratio. However, immunohistochemical analysis of four healthy bladder tissue sections found that CLEC14A protein was only detected in a single case and at levels much lower than seen in some tumours. From the analysis of *CLEC14A*/*PECAM1* ratios (see supplementary material, Table S5), there is a suggestion that *CLEC14A* gene expression is elevated in a proportion of healthy endometrial tissues. We did not detect any CLEC14A protein in samples of endometrium but we did detect CLEC14A protein, albeit at relatively low levels, in 2 of 5 healthy human placental tissues, which is consistent with the placenta being an active site of angiogenesis. *CLEC14A*/*PECAM1* ratios also suggested that *CLEC14A* transcripts may be elevated in some healthy brain tissues but this is likely to have been an artefact of delayed tissue processing of autopsy samples. Indeed, when normalising for reduced blood flow by measuring *CLEC14A*/*TIE1* ratios, there was no such concern with healthy brain tissue and we did not detect CLEC14A protein. We detected low levels of CLEC14A protein in some heart tissues but these were obtained from autopsy. Given the potential for false positives with human autopsy tissues, rapidly processed primate tissue was analysed and this showed that CLEC14A protein was undetectable in all tissues studied, including healthy heart and brain.

Thus, in general, CLEC14A expression in healthy tissues appears to be relatively low or absent. Nevertheless, if a treatment is designed to target CLEC14A, even low levels of expression in healthy tissues could potentially result in toxicity. Therefore, such treatments will need careful testing to ensure they can distinguish between different levels of target antigen expression and thereby avoid on‐target off‐tumour effects. Encouragingly, clinical studies using a CAR targeting CD22 have demonstrated that CAR T‐cells can distinguish different levels of target antigen expression in tissues [17]. CLEC14A is highly conserved between humans and animals; for example, human and mouse CLEC14A proteins show 67% amino acid sequence identity, with 78% identity within the extracellular C‐type lectin domain. Therefore, toxicity testing in healthy animal models should be most informative. In this regard, we have tested CLEC14A‐specific CAR T‐cells in healthy mice (including a knock‐in mouse that expressed the human extracellular domain of CLEC14A) and seen no evidence of toxicity, yet these CAR T‐cells inhibited tumour growth in several mouse tumour models (manuscript submitted).

The meta‐analysis of the GEO dataset indicated that *CLEC14A* transcripts may be elevated in some non‐tumour pathologies, including alcoholic hepatitis, interstitial lung disease, lung idiopathic fibrosis and muscle from gastric cancer patients. For some pathologies the number of cases analysed was again limited so it is difficult to draw firm conclusions. Equally, we were only able to test some liver and lung disease tissues for CLEC14A protein expression; although cases of hepatitis were generally negative, lower levels of CLEC14A protein were detected in some non‐cancer lung pathologies. Based on the meta‐analysis of *CLEC14A* transcripts, further protein expression studies are warranted for other non‐tumour pathologies to determine if protein expression levels could lead to unacceptable toxicity when targeting CLEC14A. Again, animal models of such pathologies may be informative when exploring the toxicity of CLEC14A‐targeted therapies. Should expression levels in some comorbidities lead to unacceptable toxicity these should be listed as exclusion criteria when designing clinical trials.

Studying protein expression in tumour tissues, we found that kidney tumours, and especially the most common type, clear cell carcinoma, expressed very high levels of CLEC14A protein. Interestingly although this elevated expression was indicated from transcript analysis using *CLEC14A*/*PECAM1* ratios, it was not from *CLEC14A*/*TIE1* ratios, implying that in this tumour elevated CLEC14A expression may be more a result of reduced flow rates. It also highlights the need to confirm transcript expression analyses with protein studies.

While a large percentage of tumours have elevated CLEC14A in their endothelium relative to healthy tissues, it is also clear from our analysis of both transcript and protein expression that not all tumours express CLEC14A. This means that CLEC14A targeted therapies would require confirmation of CLEC14A expression in the patient's tumour prior to therapy to avoid treating patients who would not respond. While biopsies are perfectly suited for such diagnostic assessment in most cases, the variability of tumour vasculature in biopsies will require stringent controls or multiple sampling from the same patient to avoid false negatives. CLEC14A levels should also be measured together with other endothelial markers and markers of reduced sheer stress to eliminate both the false negative findings (due to lack of vasculature in the biopsy sample) and false positives (due to delayed tissue processing).

## Author contributions statement

JR, KW and EJ carried out experiments. JR, KW, ZN, RB and SL analysed data. SL and RB designed the study. JR, ZN, RB and SL drafted the manuscript and all authors revised it critically. All authors had final approval of the submitted and published versions.

## Supporting information


**Supplementary materials and methods**

**Figure S1.** CLEC14A and other endothelial marker expression in healthy tissues (GeneAtlas U133A)
**Figure S2.** Defining reference intervals and exploring variability of *CLEC14A* and endothelial marker gene expression in healthy tissues
**Figure S3.** Comparative expression of endothelial markers and *CLEC14A* in tumour biopsies from the same patient
**Figure S4.** Changes in *Vimentin*, *CLEC14A*/*TIE1* and *CLEC14A*/*PECAM1* expression levels in renal cell carcinoma samples processed at different temperatures after various time periods

**Table S1.** Samples included in the meta‐analysis
**Table S2.** Number of samples of different tissue types and diagnoses
**Table S3.** Flow‐related gene expression in tissues of different origin
**Table S4.** *CLEC14A*/*TIE1* ratio in healthy tissues and cancer
**Table S5.** *CLEC14A*/*PECAM1* ratio in healthy tissues and cancer
**Table S6.** *CLEC14A*/*TIE1* ratio in non‐tumour pathologies

## Data Availability

Array‐based analyses were conducted using the GEO database (Gene Expression Omnibus, http://www.ncbi.nlm.nih.gov/geo/). The 133 studies within this database that were analysed are detailed in supplementary material, Table S1.

## References

[1] Mura M , Swain RK , Zhuang X , *et al* Identification and angiogenic role of the novel tumor endothelial marker CLEC14A. Oncogene 2012; 31: 293–305. 21706054 10.1038/onc.2011.233

[2] Pircher A , Fiegl M , Untergasser G , *et al* Favorable prognosis of operable non‐small cell lung cancer (NSCLC) patients harboring an increased expression of tumor endothelial markers (TEMs). Lung Cancer 2013; 81: 252–258. 23664449 10.1016/j.lungcan.2013.04.014

[3] Wragg JW , Durant S , McGettrick HM , *et al* Shear stress regulated gene expression and angiogenesis in vascular endothelium. Microcirculation 2014; 21: 290–300. 24471792 10.1111/micc.12119

[4] Bocci M , Sjolund J , Kurzejamska E , *et al* Activin receptor‐like kinase 1 is associated with immune cell infiltration and regulates CLEC14A transcription in cancer. Angiogenesis 2019; 22: 117–131. 30132150 10.1007/s10456-018-9642-5 PMC6510886

[5] Noy PJ , Lodhia P , Khan K , *et al* Blocking CLEC14A‐MMRN2 binding inhibits sprouting angiogenesis and tumour growth. Oncogene 2015; 34: 5821–5831. 25745997 10.1038/onc.2015.34 PMC4724939

[6] Parkhurst MR , Yang JC , Langan RC , *et al* T cells targeting carcinoembryonic antigen can mediate regression of metastatic colorectal cancer but induce severe transient colitis. Mol Ther 2011; 19: 620–626. 21157437 10.1038/mt.2010.272 PMC3048186

[7] Morgan RA , Yang JC , Kitano M , *et al* Case report of a serious adverse event following the administration of T cells transduced with a chimeric antigen receptor recognizing ERBB2. Mol Ther 2010; 18: 843–851. 20179677 10.1038/mt.2010.24 PMC2862534

[8] Lamers CH , Sleijfer S , Vulto AG , *et al* Treatment of metastatic renal cell carcinoma with autologous T‐lymphocytes genetically retargeted against carbonic anhydrase IX: first clinical experience. J Clin Oncol 2006; 24: e20–e22. 16648493 10.1200/JCO.2006.05.9964

[9] Linette GP , Stadtmauer EA , Maus MV , *et al* Cardiovascular toxicity and titin cross‐reactivity of affinity‐enhanced T cells in myeloma and melanoma. Blood 2013; 122: 863–871. 23770775 10.1182/blood-2013-03-490565 PMC3743463

[10] Morgan RA , Chinnasamy N , Abate‐Daga D , *et al* Cancer regression and neurological toxicity following anti‐MAGE‐A3 TCR gene therapy. J Immunother 2013; 36: 133–151. 23377668 10.1097/CJI.0b013e3182829903 PMC3581823

[11] Woo KV , Qu X , Babaev VR , *et al* Tie1 attenuation reduces murine atherosclerosis in a dose‐dependent and shear stress‐specific manner. J Clin Invest 2011; 121: 1624–1635. 21383501 10.1172/JCI42040 PMC3069759

[12] Porat RM , Grunewald M , Globerman A , *et al* Specific induction of tie1 promoter by disturbed flow in atherosclerosis‐prone vascular niches and flow‐obstructing pathologies. Circ Res 2004; 94: 394–401. 14670840 10.1161/01.RES.0000111803.92923.D6

[13] Chen‐Konak L , Guetta‐Shubin Y , Yahav H , *et al* Transcriptional and post‐translation regulation of the Tie1 receptor by fluid shear stress changes in vascular endothelial cells. FASEB J 2003; 17: 2121–2123. 14500555 10.1096/fj.02-1151fje

[14] Kuzu I , Bicknell R , Harris AL , *et al* Heterogeneity of vascular endothelial cells with relevance to diagnosis of vascular tumours. J Clin Pathol 1992; 45: 143–148. 1371777 10.1136/jcp.45.2.143 PMC495659

[15] Givens C , Tzima E . Endothelial mechanosignaling: does one sensor fit all? Antioxid Redox Signal 2016; 25: 373–388. 27027326 10.1089/ars.2015.6493 PMC5011625

[16] Liu NW , Sanford T , Srinivasan R , *et al* Impact of ischemia and procurement conditions on gene expression in renal cell carcinoma. Clin Cancer Res 2013; 19: 42–49. 23136194 10.1158/1078-0432.CCR-12-2606 PMC3658320

[17] Fry TJ , Shah NN , Orentas RJ , *et al* CD22‐targeted CAR T cells induce remission in B‐ALL that is naive or resistant to CD19‐targeted CAR immunotherapy. Nat Med 2018; 24: 20–28. 29155426 10.1038/nm.4441 PMC5774642

[18] CLSI . C28‐A3 document; Defining, establishing and verifying reference intervals in the clinical laboratory: approved guideline (3rd edn). CLSI, 2008; 1–76.

[19] Efron B , Tibshirani RJ . An introduction to the Bootstrap (1st edn). Chapman and Hall/CRC: Boca Raton, FL, 1994.

