# Supplementary material for: An evaluation of the tumour endothelial marker CLEC14A as a therapeutic target in solid tumours
Source: J Pathol Clin Res. 2020 Jul 21;6(4):308–19. doi: 10.1002/cjp2.176 (PMC7578301; doi:10.1002/cjp2.176)
Supplement: Supplementary file 1 — Supplementary materials and methods [file CJP2-6-308-s001.docx]

**An evaluation of the tumour endothelial marker CLEC14A as a therapeutic target in solid tumours**

Robinson J *et al*. *J Pathol Clin Res* DOI: 10.1002/cjp2.176

**Supplementary materials and methods**

**Reference numbers refer to the list in the main text**

**Analysis of public databases for *CLEC14A* transcript expression in healthy and cancer tissues**

The expression pattern of *CLEC14A* was studied in a collection of healthy tissues (GeneAtlas U133A, gcrma, <http://biogps.org>). From the 176 samples in this dataset those containing tumours or microdissected or isolated cells were excluded leaving a total of 118 healthy tissue samples. To determine the expected range of expression of endothelial markers within healthy tissues (reference intervals) we used the "robust method" as described in the Clinical and Laboratory Standards Institute's (CLSI) Guidelines C28-A3 [18]. With this method, the confidence intervals for the reference limits were then estimated using bootstrapping [19]. Ten thousand iterations were performed to increase the reliability of the confidence intervals of the calculated reference value. For each variable the reference interval was set to 95%. Outliers were not removed from the analysis. Regression analysis was used to evaluate the relationship between different endothelial markers.

Collecting data from the GEO database (Gene Expression Omnibus, [*http://www.ncbi.nlm.nih.gov/geo/*](http://www.ncbi.nlm.nih.gov/geo/)), the primary inclusion criterion was the presence of *CLEC14A* and *ACTB* expression values in human tissue samples. Studies using microdissected or isolated cells, and in vitro studies (cell lines, tumour explants, etc) were excluded. This led to the identification of 133 studies. Tissue samples were then grouped based on diagnosis. Healthy tissues were then separated into two subgroups depending on whether they came from completely healthy donors or healthy adjacent to diseased (tumour or otherwise) tissue. Tumours were subdivided into primary untreated, primary treated and metastatic tumours. From each dataset, besides the diagnosis and tissue type, the expression profile of *CLEC14A*, *PECAM*, *vWF*, *TIE1* and *ACTB* were downloaded. Log expression values for each transcript of interest were then normalised to *ACTB*. Samples with tissue vascularity (as measured by *PECAM1*/*ACTB* and *vWF*/*ACTB* ratio) below the lower limit of the previously defined reference intervals in healthy tissue were considered to lack informative vasculature and therefore excluded from the study. Fetal tissue samples were also excluded from the study.

**Immunohistochemistry: human tissue**

Tissue sections were dewaxed in xylene and isopropanol. Endogenous peroxidase activity was blocked by 5 min incubation in a 3% hydrogen peroxide, 90% methanol solution. High pH antigen unmasking solution (Vector Laboratories, Peterborough, UK) was heated in an 850W microwave oven for 5 min; the TMAs were placed into the solution and heated for a further 15 min, before being left to cool for 10 min. Non-specific antibody interactions were then blocked by 30 min incubation with 10% horse serum in PBS.

For staining of CD31, the TMAs were incubated for 18 hr at 4°C with primary antibody (dilution 1:40; JC70A; Dako, Santa Clara CA., USA), followed by ImmPRESS Reagent Peroxidase Anti-Mouse (dilution 1:4; Vector laboratories) for 3 hr at 4°C. For staining of CLEC14A the TMAs were incubated with a polyclonal primary antibody (0.85µg/ml; AF4968; R&D systems, Minneapolis MIN., United States) or concentration matched isotype control (5-001-A; R&D) for 1 hr at 23°C, followed by anti-sheep HRP (dilution 1:100; HAF016; R&D) for 1hr at 23°C.

**Immunohistochemistry: Cynomolgus macaque tissue**

Tissue was fixed in -30°C acetone for 10 min, before being blocked for 10 min with BLOXALL solution and for 20 min with 2.5% horse serum. The tissue sections were then incubated at 23°C with either an anti-CLEC14A monoclonal antibody (25 ng/ml; CRT3) or an anti CD31 polyclonal antibody (1:160; JC70A; Dako) for 2.5 hours. The sections were then incubated with Amplifier Antibody for 15 min followed by ImmPRESS Excel reagent for 30 min.

**Development and imaging**

Immunohistochemical staining was developed using an ImmPACT NovaRED Peroxidase substrate kit (Vector), incubating at 23°C for 3mins. Tissues were then counterstained with haematoxylin before being dehydrated and mounted with cover slips. Images were acquired using a Zeiss Axioskop 40 microscope and Zeiss EC Plan-NEOFLUAR 10X objective.
