# Supplementary material for: An evaluation of the tumour endothelial marker CLEC14A as a therapeutic target in solid tumours
Source: J Pathol Clin Res. 2020 Jul 21;6(4):308–19. doi: 10.1002/cjp2.176 (PMC7578301; doi:10.1002/cjp2.176)
Supplement: Supplementary file 2 — Figure S1. CLEC14A and other endothelial marker expression in healthy tissues (GeneAtlas U133A) Figure S2. Defining reference intervals and exploring variability of CLEC14A and endothelial marker gene expression in healthy tissues Figure S3. Comparative expression of endothelial markers and CLEC14A in tumour biopsies from the same patient Figure S4. Changes in Vimentin, CLEC14A/TIE1 and CLEC14A/PECAM1 expression levels in renal cell carcinoma samples processed at different temperatures after various time periods [file CJP2-6-308-s002.docx]

**An evaluation of the tumour endothelial marker CLEC14A as a therapeutic target in solid tumours**

Robinson J *et al*. *J Pathol Clin Res* DOI: 10.1002/cjp2.176

**Supplementary Figures**

**
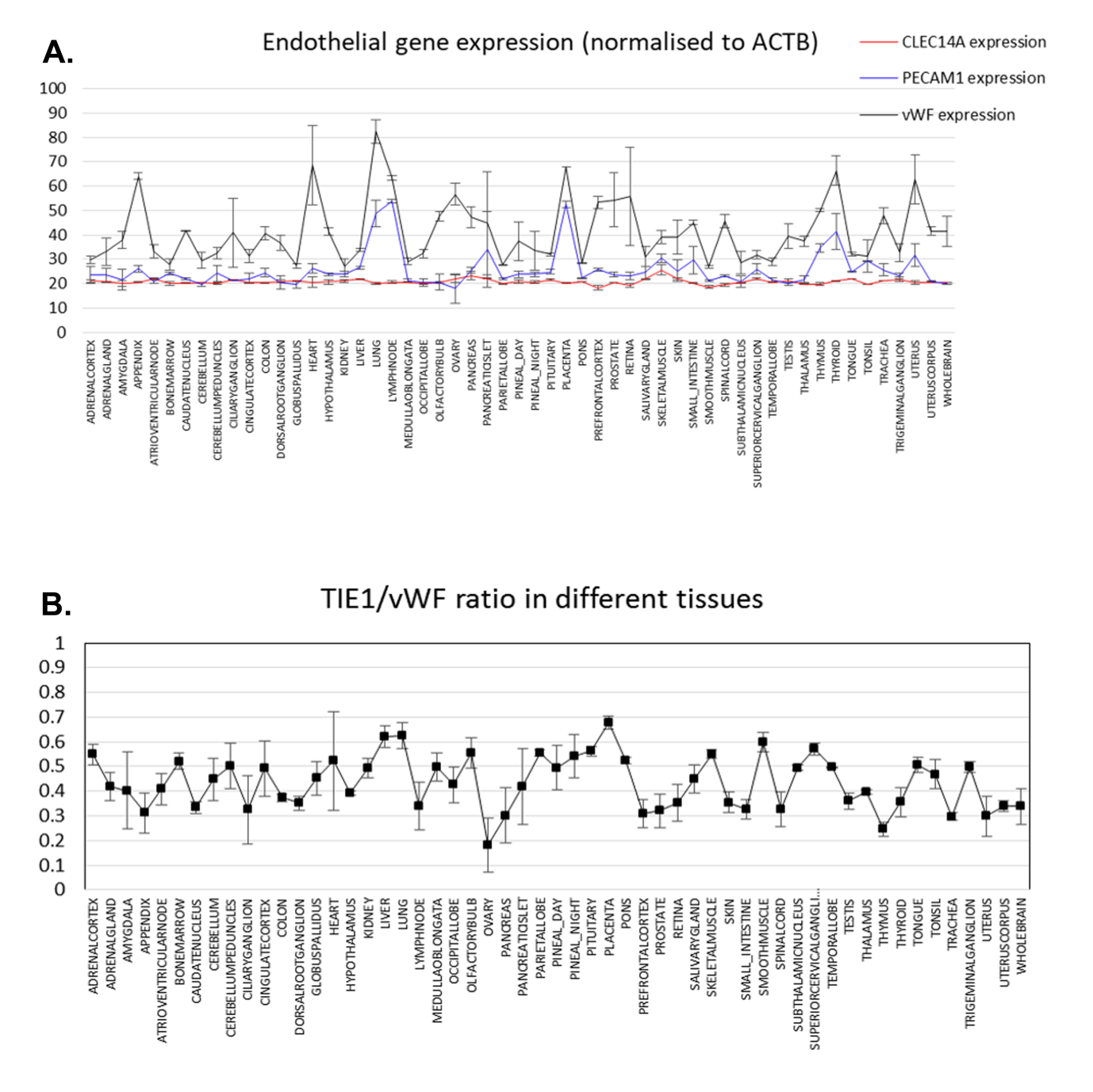
**

**Figure S1.** *CLEC14A* and other endothelial marker expression in healthy tissues (GeneAtlas U133A). (A) Relative expression (normalised to *ACTB*) of three endothelial markers in healthy tissue samples from various organs. The y axis shows log *ACTB* normalised expression of *CLEC14A* (red), *PECAM1* (blue) and *vWF* (black) expressed as a percentage. (B) *TIE1*/*vWF* ratio in healthy tissues. Y axis represents the ratio of the two markers. Data points represent mean values and error bars indicate the standard deviation.

**
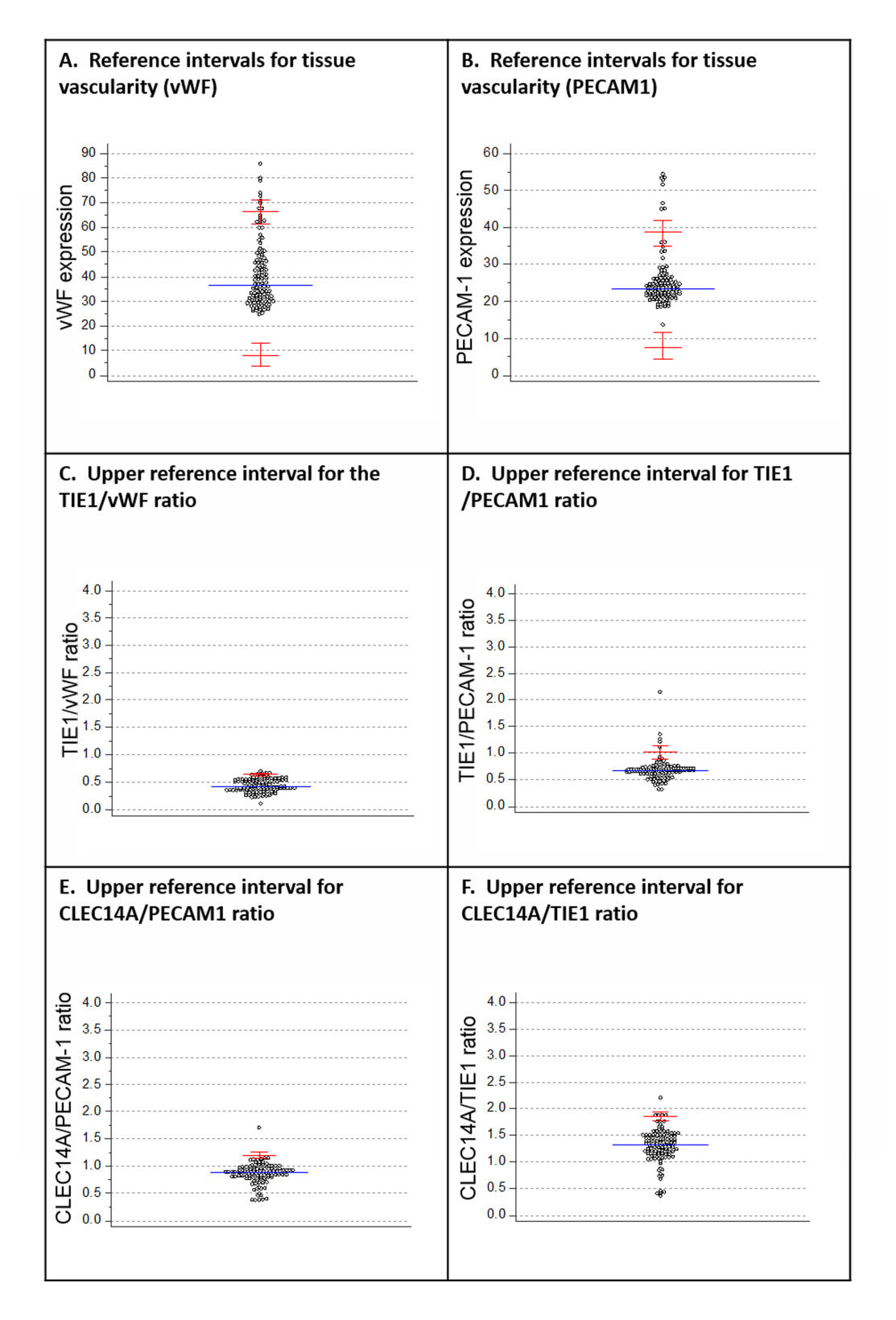
**

**Figure S2.** Defining reference intervals and exploring variability of *CLEC14A* and endothelial marker gene expression in healthy tissues. (A-B) Upper and lower reference intervals are indicated in red for *vWF* and *PECAM-1* expression in healthy tissues. The y axis represents the log *ACTB* normalised expression of these endothelial markers. (C-F) Upper reference intervals for the ratios of different endothelial markers used in the study. The y axis shows the ratio of gene expression. Data from GeneAtlas U133A.


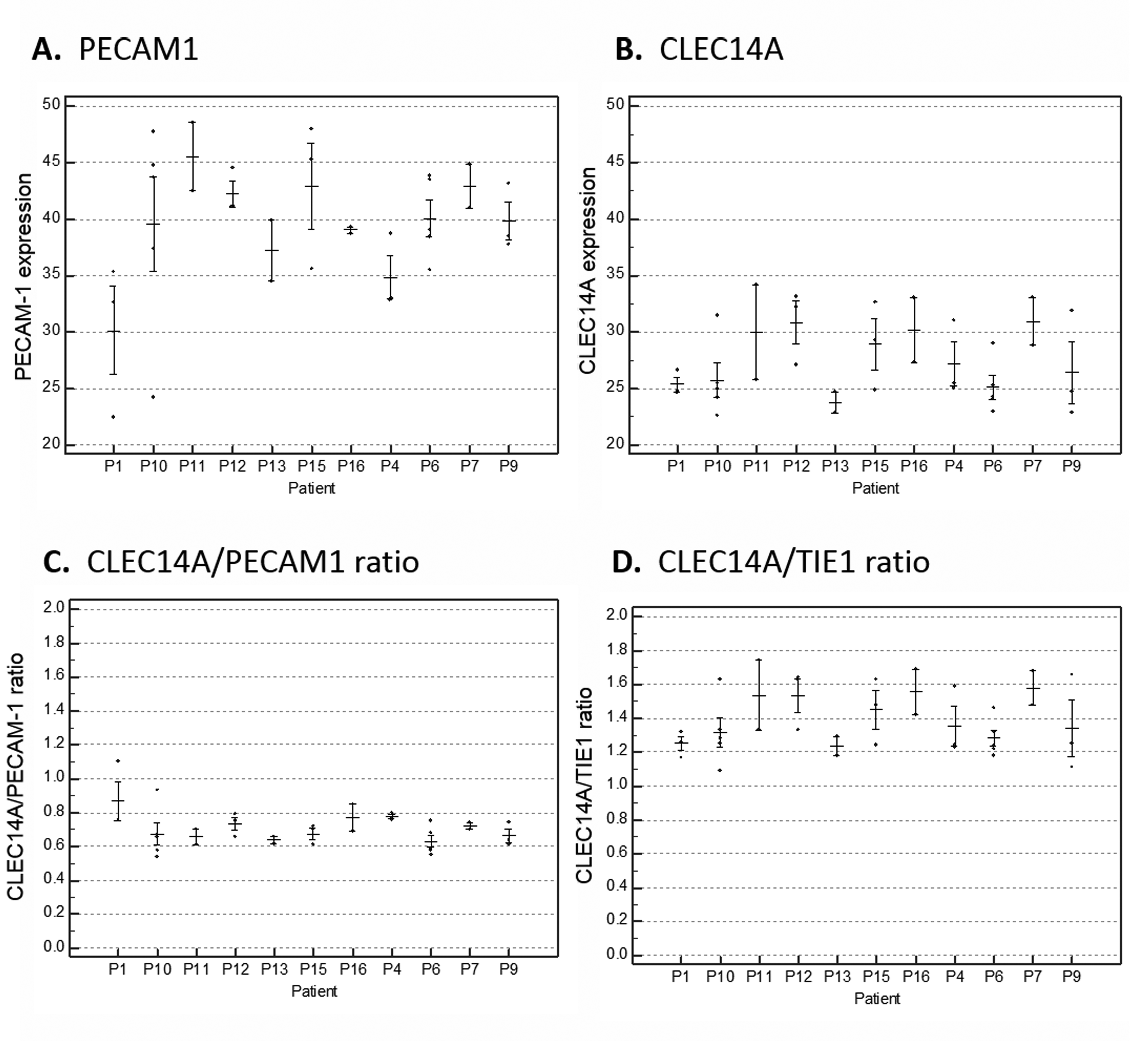


**Figure S3.** Comparative expression of endothelial markers and *CLEC14A* in tumour biopsies from the same patient. Log expression (normalised to *ACTB*) of (A) *PECAM-1* (B) *CLEC14A* (C) *CLEC14A*/*PECAM-1* ratio and (D) *CLEC14A*/*TIE1* ratio in tumour biopsies. Data points shown are for each tumour biopsy taken from a patient (with means and standard deviations for each patient). Data taken from GEO dataset study GDS4547.

**
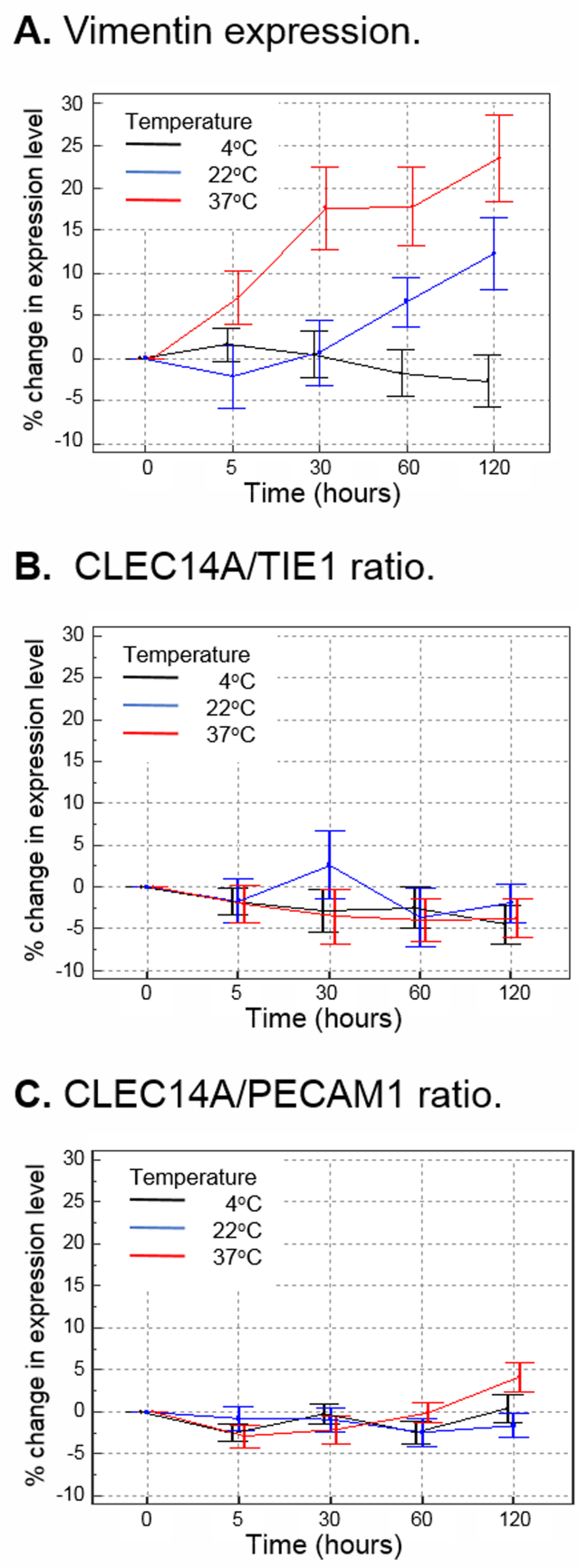
**

**Figure S4.** Changes in *Vimentin*, *CLEC14A*/*TIE1* and *CLEC14A*/*PECAM1* expression levels in renal cell carcinoma samples processed at different temperatures after various time periods. The y axis represents the percentage change in expression relative to the sample processed at the 0 hour time-point. Data points represent mean values and error bars indicate the standard error of the mean.
