# Supplementary material for: An evaluation of the tumour endothelial marker CLEC14A as a therapeutic target in solid tumours
Source: J Pathol Clin Res. 2020 Jul 21;6(4):308–19. doi: 10.1002/cjp2.176 (PMC7578301; doi:10.1002/cjp2.176)
Supplement: Supplementary file 3 — Table S1. Samples included in the meta‐analysis Table S2. Number of samples of different tissue types and diagnoses Table S3. Flow‐related gene expression in tissues of different origin Table S4. CLEC14A/TIE1 ratio in healthy tissues and cancer Table S5. CLEC14A/PECAM1 ratio in healthy tissues and cancer Table S6. CLEC14A/TIE1 ratio in non‐tumour pathologies [file CJP2-6-308-s003.docx]

**An evaluation of the tumour endothelial marker CLEC14A as a therapeutic target in solid tumours**

Robinson J *et al*. *J Pathol Clin Res* DOI: 10.1002/cjp2.176

**Supplementary Tables**

**Table S1**. Samples included in the meta-analysis.

| **Study** | **Healthy tissue** | **Healthy tissue near pathology** | **Healthy tissue near tumour** | **Primary tumour untreated** | **Metastasis** | **Non-tumour pathology** | **Post-treatment tumour** | **Repeat sampling of tumour tissue** | **Total** |
| --- | --- | --- | --- | --- | --- | --- | --- | --- | --- |
| GDS1263 |  |  |  | 15 |  |  |  |  | 15 |
| GDS1344 |  |  |  | 34 |  |  |  |  | 34 |
| GDS1439 |  |  |  | 13 |  | 6 |  |  | 19 |
| GDS1665 |  |  | 9 | 9 |  |  |  |  | 18 |
| GDS1673 | 40 |  |  |  |  |  |  |  | 40 |
| GDS1732 |  |  | 7 | 7 |  |  |  |  | 14 |
| GDS1989 | 2 |  |  | 9 |  | 4 |  |  | 15 |
| GDS2052 | 27 |  |  |  |  |  |  |  | 27 |
| GDS2083 | 5 |  |  |  |  | 5 |  |  | 10 |
| GDS2089 |  |  |  |  |  | 6 |  |  | 6 |
| GDS2250 | 7 |  |  | 40 |  |  |  |  | 47 |
| GDS2416 |  |  |  | 33 |  |  |  |  | 33 |
| GDS2609 | 10 |  |  |  |  | 12 |  |  | 22 |
| GDS2737 | 16 |  |  |  |  | 21 |  |  | 37 |
| GDS2835 |  | 10 |  |  |  | 10 |  |  | 20 |
| GDS2881 |  |  | 10 | 10 |  |  |  |  | 20 |
| GDS2935 | 16 | 4 |  |  |  | 14 |  |  | 34 |
| GDS2947 |  |  | 32 |  |  | 32 |  |  | 64 |
| GDS3100 | 20 |  |  |  |  | 10 |  |  | 30 |
| GDS3104 | 13 |  |  |  |  | 16 |  |  | 29 |
| GDS3113 | 81 |  |  |  |  |  |  |  | 81 |
| GDS3141 |  | 19 |  |  |  |  |  |  | 19 |
| GDS3182 | 36 |  |  |  |  |  |  |  | 36 |
| GDS3268 |  |  |  |  |  | 2 |  |  | 2 |
| GDS3274 |  |  |  | 18 |  |  |  |  | 18 |
| GDS3313 | 31 |  |  |  |  |  |  |  | 31 |
| GDS3423 | 8 |  |  |  |  |  |  |  | 8 |
| GDS3463 | 8 |  |  |  |  | 13 |  |  | 21 |
| GDS3501 |  |  |  |  | 18 |  |  |  | 18 |
| GDS3539 | 21 | 28 |  |  |  | 33 |  |  | 82 |
| GDS3580 | 6 |  |  |  |  | 6 |  |  | 12 |
| GDS3627 |  |  |  | 58 |  |  |  |  | 58 |
| GDS3630 | 40 |  |  |  |  |  |  |  | 40 |
| GDS3705 |  |  |  |  |  | 15 |  |  | 15 |
| GDS3756 |  |  | 10 | 13 |  | 10 | 9 |  | 42 |
| GDS3762 | 21 |  |  |  |  |  |  |  | 21 |
| GDS3806 | 7 | 9 |  |  |  |  |  |  | 16 |
| GDS3837 |  |  | 60 | 60 |  |  |  |  | 120 |
| GDS3853 | 5 |  |  | 14 |  |  |  |  | 19 |
| GDS3855 | 11 | 11 |  |  |  |  |  |  | 22 |
| GDS3880 | 12 | 30 |  |  |  |  |  |  | 42 |
| GDS3884 | 40 | 10 |  |  |  |  |  |  | 50 |
| GDS3897 |  |  |  |  |  | 43 |  |  | 43 |
| GDS3951 |  |  | 6 |  |  | 23 |  |  | 29 |
| GDS3975 | 9 |  |  |  |  | 10 |  |  | 19 |
| GDS3981 |  |  |  |  |  | 20 |  |  | 20 |
| GDS4053 |  |  |  | 43 |  |  |  |  | 43 |
| GDS4069 |  |  |  | 19 |  |  |  |  | 19 |
| GDS4093 |  |  |  | 11 |  |  | 11 |  | 22 |
| GDS4102 |  |  | 16 | 36 |  |  |  |  | 52 |
| GDS4103 |  |  | 39 | 39 |  |  |  |  | 78 |
| GDS4133 |  |  |  |  |  | 23 |  |  | 23 |
| GDS4176 |  |  |  | 36 |  |  |  |  | 36 |
| GDS4198 |  |  |  | 70 |  |  |  |  | 70 |
| GDS4218 | 2 |  |  |  |  | 5 |  |  | 7 |
| GDS4221 |  |  |  | 15 |  |  |  |  | 15 |
| GDS4222 |  |  |  | 130 |  |  |  |  | 130 |
| GDS4279 | 6 |  |  |  |  | 17 |  |  | 23 |
| GDS4281 |  |  |  | 29 |  |  |  |  | 29 |
| GDS4282 |  |  | 23 | 29 |  |  |  |  | 52 |
| GDS4314 |  | 5 |  |  |  | 19 |  |  | 24 |
| GDS4318 | 108 |  |  |  |  |  |  |  | 108 |
| GDS4336 |  |  | 45 | 45 |  |  |  |  | 90 |
| GDS4337 | 54 |  |  |  |  | 9 |  |  | 63 |
| GDS4345 | 6 |  |  |  |  | 24 |  |  | 30 |
| GDS4350 |  | 8 |  |  |  | 20 |  |  | 28 |
| GDS4354 |  |  |  |  |  | 18 |  |  | 18 |
| GDS4358 | 18 |  |  |  |  | 54 |  |  | 72 |
| GDS4365 | 13 |  |  |  |  | 30 |  |  | 43 |
| GDS4374 |  |  |  |  |  | 16 |  |  | 16 |
| GDS4379 |  |  |  | 62 |  |  |  |  | 62 |
| GDS4381 |  |  |  | 27 |  |  |  |  | 27 |
| GDS4382 |  |  | 17 | 17 |  |  |  |  | 34 |
| GDS4384 |  |  |  | 10 |  |  |  |  | 10 |
| GDS4389 | 7 |  |  |  |  | 15 |  |  | 22 |
| GDS4393 |  |  |  | 33 | 21 |  |  |  | 54 |
| GDS4396 |  |  |  | 23 | 6 |  |  |  | 29 |
| GDS4404 | 24 |  |  |  |  | 26 |  |  | 50 |
| GDS4412 | 56 |  |  |  |  |  |  |  | 56 |
| GDS4426 | 6 |  |  |  |  | 6 |  |  | 12 |
| GDS4444 |  | 8 |  |  |  | 27 |  |  | 35 |
| GDS4456 |  |  |  | 93 |  |  |  |  | 93 |
| GDS4460 |  |  |  |  |  | 30 |  |  | 30 |
| GDS4464 |  |  |  | 34 |  |  |  |  | 34 |
| GDS4465 |  |  |  | 26 |  |  |  |  | 26 |
| GDS4467 |  |  | 2 | 30 |  |  |  |  | 32 |
| GDS4469 |  |  |  | 19 |  |  |  |  | 19 |
| GDS4470 |  |  |  | 46 |  |  |  |  | 46 |
| GDS4471 |  |  |  | 76 |  |  |  |  | 76 |
| GDS4477 |  |  |  | 27 |  |  |  |  | 27 |
| GDS4491 | 8 | 12 |  |  |  | 13 |  |  | 33 |
| GDS4513 |  |  |  | 53 |  |  |  |  | 53 |
| GDS4519 | 10 |  |  |  |  | 10 |  |  | 20 |
| GDS4522 | 18 |  |  |  |  | 22 |  |  | 40 |
| GDS4523 | 23 |  |  |  |  | 28 |  |  | 51 |
| GDS4547 |  |  |  | 10 |  |  |  | 130 | 140 |
| GDS4589 | 12 |  |  | 91 |  |  |  |  | 103 |
| GDS4600 |  | 85 |  |  |  | 85 |  |  | 170 |
| GDS4664 |  |  |  | 39 |  |  |  |  | 39 |
| GDS4758 | 47 |  |  |  |  | 32 |  |  | 79 |
| GDS4760 |  |  |  | 10 |  |  |  |  | 10 |
| GDS4761 |  |  |  |  | 90 |  |  |  | 90 |
| GDS4772 | 5 |  |  |  |  | 12 |  |  | 17 |
| GDS4794 | 38 |  |  | 23 |  |  |  |  | 61 |
| GDS4820 | 45 |  |  |  |  |  |  |  | 45 |
| GDS4824 |  |  | 8 | 13 |  |  |  |  | 21 |
| GDS4838 |  |  |  | 23 |  |  |  |  | 23 |
| GDS4841 | 5 |  |  |  |  | 14 |  |  | 19 |
| GDS4858 | 22 |  |  |  |  |  |  |  | 22 |
| GDS4879 | 19 |  |  |  |  | 20 |  |  | 39 |
| GDS4881 | 41 |  |  |  |  | 32 |  |  | 73 |
| GDS4887 |  | 20 |  | 20 |  |  |  |  | 40 |
| GDS4894 | 14 |  |  |  |  |  |  |  | 14 |
| GDS4906 | 24 |  |  |  |  | 30 |  |  | 54 |
| GDS4909 | 12 |  |  |  |  | 12 |  |  | 24 |
| GDS4923 |  | 27 |  |  |  |  |  |  | 27 |
| GDS4950 |  |  |  | 28 |  |  |  |  | 28 |
| GDS4975 |  |  |  | 81 |  |  |  |  | 81 |
| GDS5027 |  |  |  | 156 |  |  |  |  | 156 |
| GDS507 |  |  | 8 | 9 |  |  |  |  | 17 |
| GDS5204 | 41 |  |  |  |  |  |  |  | 41 |
| GDS5216 | 36 |  |  |  |  |  |  |  | 36 |
| GDS5218 | 110 |  |  |  |  |  |  |  | 110 |
| GDS5272 |  | 5 |  |  |  | 5 |  |  | 10 |
| GDS5306 |  |  |  | 19 | 19 |  |  |  | 38 |
| GDS5338 | 3 |  |  |  |  | 6 |  |  | 9 |
| GDS5362 | 4 |  |  | 5 |  |  |  |  | 9 |
| GDS5392 |  | 4 |  |  |  | 4 |  |  | 8 |
| GDS5411 | 3 |  |  |  |  | 6 |  |  | 9 |
| GDS5420 |  | 24 |  |  |  | 75 |  |  | 99 |
| GDS5473 | 48 |  |  |  |  |  |  |  | 48 |
| GDS5610 | 2 |  |  |  |  | 4 |  |  | 6 |
| GDS651 | 11 |  |  |  |  | 26 |  |  | 37 |
| **Grand Total** | **1393** | **319** | **292** | **1938** | **154** | **1086** | **20** | **130** | **5332** |

**Table S2.** Number of samples of different tissue types and diagnoses.

| **Tissue** | **Healthy tissue** | **Healthy tissue near pathology** | **Healthy tissue near tumour** | **Primary tumour untreated** | **Metastasis** | **Non-tumour pathology** | **Post-treatment tumour** | **Repeat sampling of tumour tissue** | **Total** |
| --- | --- | --- | --- | --- | --- | --- | --- | --- | --- |
| Bladder | 1 |  |  | 93 |  |  |  |  | 94 |
| Bone |  |  |  |  | 5 | 18 |  |  | 23 |
| Bone marrow | 4 |  |  | 19 |  |  |  |  | 23 |
| Brain | 178 |  | 2 | 281 | 19 | 161 |  |  | 641 |
| Breast | 16 |  |  | 312 | 11 |  | 11 |  | 350 |
| Cervix |  |  |  | 72 |  |  |  |  | 72 |
| Colon | 77 | 19 | 59 | 253 | 27 | 155 | 9 |  | 599 |
| Endometrium | 95 | 37 |  | 91 |  | 41 |  |  | 264 |
| Oesophagus |  | 8 |  |  |  | 10 |  |  | 18 |
| Heart | 130 | 5 |  |  |  | 61 |  |  | 196 |
| Kidney | 4 |  | 41 | 110 |  |  |  | 130 | 285 |
| Liver | 60 | 20 |  | 20 | 34 | 47 |  |  | 181 |
| Lung | 56 |  | 66 | 141 | 2 | 61 |  |  | 326 |
| Lymph node | 1 |  |  | 246 | 39 |  |  |  | 286 |
| Muscle | 572 | 51 |  |  |  | 199 |  |  | 822 |
| Other | 54 |  |  |  |  |  |  |  | 54 |
| Ovary | 4 |  |  | 28 |  |  |  |  | 32 |
| Pancreas | 58 |  | 100 | 120 |  | 9 |  |  | 287 |
| Prostate | 4 |  | 8 | 26 |  | 6 |  |  | 44 |
| Skin | 64 | 179 |  | 6 | 17 | 296 |  |  | 562 |
| Stomach | 7 |  |  | 70 |  | 22 |  |  | 99 |
| Thyroid | 8 |  | 16 | 21 |  |  |  |  | 45 |
| Uvea |  |  |  | 29 |  |  |  |  | 29 |
| **Grand Total** | **1393** | **319** | **292** | **1938** | **154** | **1086** | **20** | **130** | **5332** |

**Table S3.** Flow-related gene expression in tissues of different origin

| **Tissue collection type** | **Number of samples where *TIE1* expression data not available** | **Number of samples with Normal Flow^** | **Number of samples with Stasis^** | **Total number of samples** |
| --- | --- | --- | --- | --- |
| Post Mortem |  | 165 | 513 | 678 |
| Surgical | 37 | 2452 | 1455 | 3944 |
| Surgical* | 2 | 474 | 234 | 710 |
| **Grand Total** | **39** | **3091** | **2202** | **5332** |

*Healthy tissues taken from site adjacent to the primary pathology (tumour or otherwise)

^*TIE1*/*vWF* ratio<0.7 was regarded as an indicator of normal flow and *TIE1*/*vWF* ratio>0.7 as stasis (based on data shown in supplementary material, Figure S1B)

**Table S4.** *CLEC14A*/*TIE1* ratio in healthy tissues and cancer

| Tissue | Number of samples | | % samples above 69% of Controls* | | % samples above 87% of Controls | | % samples above 95% of Controls | |
| --- | --- | --- | --- | --- | --- | --- | --- | --- |
|  | Healthy | Tumour | Healthy | Tumour | Healthy | Tumour | Healthy | Tumour |
| Bladder | 1 | 93 | 100.0 | 100.0 | 100.0 | 98.9 | 100.0 | 95.7 |
| Bone | 0 | 5 | N/A | 80.0 | N/A | 60.0 | N/A | 20.0 |
| Bone marrow | 4 | 19 | 25.0 | 31.6 | 25.0 | 31.6 | 25.0 | 31.6 |
| Brain | 178 | 300 | 3.9 | 10.7 | 2.8 | 6.7 | 2.3 | 4.0 |
| Breast | 16 | 323 | 81.3 | 80.2 | 81.3 | 64.4 | 75.0 | 31.0 |
| Cervix | 0 | 72 | N/A | 70.8 | N/A | 65.3 | N/A | 37.5 |
| Colon | 77 | 280 | 15.6 | 86.1 | 7.8 | 65.7 | 5.2 | 34.6 |
| Endometrium | 95 | 91 | 11.6 | 62.6 | 9.5 | 53.9 | 5.3 | 41.8 |
| Heart | 130 | 0 | 8.5 | N/A | 7.7 | N/A | 3.9 | N/A |
| Kidney | 4 | 110 | 25.0 | 9.1 | 25.0 | 6.4 | 25.0 | 4.6 |
| Liver | 60 | 54 | 11.7 | 48.2 | 11.7 | 38.9 | 8.3 | 18.5 |
| Lung | 56 | 143 | 12.5 | 93.0 | 12.5 | 91.6 | 12.5 | 67.8 |
| Lymph node | 1 | 285 | 0.0 | 15.8 | 0.0 | 9.1 | 0.0 | 4.9 |
| Ovary | 4 | 28 | 25.0 | 71.4 | 25.0 | 64.3 | 25.0 | 50.0 |
| Pancreas | 58 | 120 | 3.5 | 60.0 | 1.7 | 56.7 | 1.7 | 36.7 |
| Prostate | 4 | 26 | 0.0 | 92.3 | 0.0 | 84.6 | 0.0 | 57.7 |
| Skin | 64 | 23 | 1.6 | 52.2 | 0.0 | 39.1 | 0.0 | 13.0 |
| Stomach | 7 | 70 | 14.3 | 100.0 | 14.3 | 100.0 | 14.3 | 77.1 |
| Thyroid | 8 | 21 | 12.5 | 4.8 | 12.5 | 0.0 | 12.5 | 0.0 |
| Uvea | 0 | 29 | N/A | 48.3 | N/A | 41.4 | N/A | 27.6 |
| **Total** | **767** | **2092** | **20.7** | **58.8** | **19.8** | **51.5** | **18.6** | **34.4** |

N/A = not available

*controls = healthy tissues taken from GeneAtlas U133A database

**Table S5**. *CLEC14A*/*PECAM1* ratio in healthy tissues and cancer

| Tissue | Number of samples | | % samples above 69% of Controls* | | % samples above 87% of Controls | | % samples above 95% of Controls | |
| --- | --- | --- | --- | --- | --- | --- | --- | --- |
|  | Healthy | Tumour | Healthy | Tumour | Healthy | Tumour | Healthy | Tumour |
| Bladder | 1 | 93 | 0.0 | 47.3 | 0.0 | 16.1 | 0.0 | 0.0 |
| Bone | 0 | 5 | N/A | 0.0 | N/A | 0.0 | N/A | 0.0 |
| Bone marrow | 4 | 19 | 0.0 | 0.0 | 0.0 | 0.0 | 0.0 | 0.0 |
| Brain | 178 | 300 | 41.0 | 48.3 | 28.1 | 29.0 | 3.9 | 11.0 |
| Breast | 16 | 323 | 18.8 | 41.5 | 6.3 | 28.8 | 0.0 | 12.4 |
| Cervix | 0 | 72 | N/A | 47.2 | N/A | 18.1 | N/A | 0.0 |
| Colon | 77 | 280 | 10.4 | 25.0 | 7.8 | 11.8 | 1.3 | 5.7 |
| Endometrium | 95 | 91 | 77.9 | 84.6 | 49.5 | 73.6 | 11.6 | 47.3 |
| Heart | 130 | 0 | 0.0 | N/A | 0.0 | N/A | 0.0 | N/A |
| Kidney | 4 | 110 | 0.0 | 33.6 | 0.0 | 22.7 | 0.0 | 0.0 |
| Liver | 60 | 54 | 18.3 | 7.4 | 15.0 | 1.9 | 3.3 | 0.0 |
| lung | 56 | 143 | 1.8 | 15.4 | 0.0 | 6.3 | 0.0 | 2.8 |
| Lymph node | 1 | 285 | 0.0 | 6.3 | 0.0 | 2.8 | 0.0 | 1.8 |
| Ovary | 4 | 28 | 0.0 | 53.6 | 0.0 | 28.6 | 0.0 | 10.7 |
| Pancreas | 58 | 120 | 0.0 | 3.3 | 0.0 | 0.8 | 0.0 | 0.0 |
| Prostate | 4 | 26 | 0.0 | 46.2 | 0.0 | 23.1 | 0.0 | 3.9 |
| Skin | 64 | 23 | 10.9 | 0.0 | 0.0 | 0.0 | 0.0 | 0.0 |
| Stomach | 7 | 70 | 0.0 | 12.9 | 0.0 | 1.4 | 0.0 | 0.0 |
| Thyroid | 8 | 21 | 0.0 | 66.7 | 0.0 | 42.9 | 0.0 | 9.5 |
| Uvea | 0 | 29 | N/A | 62.1 | N/A | 55.2 | N/A | 41.4 |
| Total | **767** | **2092** | **10.5** | **31.7** | **6.3** | **19.1** | **1.2** | **7.7** |

N/A = not available

*controls = healthy tissues taken from GeneAtlas U133A database

**Table S6.** *CLEC14A*/*TIE1* ratio in non-tumour pathologies

| Tissue Pathology | Number of samples | % samples above 50% of Controls* | % samples above 69% of Controls | % samples above 87% of Controls | % samples above 95% of Controls |
| --- | --- | --- | --- | --- | --- |
| Alcoholic hepatitis | 15 | 100.0 | 100.0 | 100.0 | 100.0 |
| Atrophic Stomach | 6 | 0.0 | 0.0 | 0.0 | 0.0 |
| Atypical nevus | 2 | 0.0 | 0.0 | 0.0 | 0.0 |
| Barrett oesophagus | 20 | 0.0 | 0.0 | 0.0 | 0.0 |
| Benign nevus | 2 | 0.0 | 0.0 | 0.0 | 0.0 |
| Bone Cushing Syndrome post-surgery | 9 | 0.0 | 0.0 | 0.0 | 0.0 |
| Bone Cushing Syndrome Untreated | 9 | 0.0 | 0.0 | 0.0 | 0.0 |
| Brain Alcoholism | 20 | 0.0 | 0.0 | 0.0 | 0.0 |
| Brain Alzheimer's disease | 32 | 0.0 | 0.0 | 0.0 | 0.0 |
| Brain HIV | 18 | 5.6 | 0.0 | 0.0 | 0.0 |
| Brain HIV and HIV-associated Dementia | 36 | 0.0 | 0.0 | 0.0 | 0.0 |
| Brain MS | 5 | 0.0 | 0.0 | 0.0 | 0.0 |
| Brain Schizophrenia | 50 | 4.0 | 2.0 | 2.0 | 0.0 |
| Colon Inflammatory bowel disease | 24 | 54.2 | 45.8 | 45.8 | 25.0 |
| Colon Irritable bowel syndrome | 19 | 47.4 | 47.4 | 31.6 | 5.3 |
| Colon Parasitic infection | 16 | 0.0 | 0.0 | 0.0 | 0.0 |
| Colon Ulcerative colitis | 27 | 74.1 | 48.2 | 40.7 | 18.5 |
| Colon Ulcerative Colitis active non invaded mucosa | 7 | 85.7 | 14.3 | 0.0 | 0.0 |
| Colon Ulcerative Colitis remission | 8 | 87.5 | 12.5 | 0.0 | 0.0 |
| Dermatomyositis | 5 | 40.0 | 0.0 | 0.0 | 0.0 |
| Diabetic heart failure | 7 | 0.0 | 0.0 | 0.0 | 0.0 |
| Dilated cardiomyopathy | 29 | 48.3 | 41.4 | 20.7 | 17.2 |
| Endometrioma | 10 | 0.0 | 0.0 | 0.0 | 0.0 |
| Endometriosis | 31 | 0.0 | 0.0 | 0.0 | 0.0 |
| Facioscapulohumeral muscular dystrophy | 26 | 0.0 | 0.0 | 0.0 | 0.0 |
| Helicobacter infected | 6 | 0.0 | 0.0 | 0.0 | 0.0 |
| Inclusion body myositis | 4 | 25.0 | 0.0 | 0.0 | 0.0 |
| Interstitial lung diseases | 23 | 100.0 | 100.0 | 100.0 | 100.0 |
| Ischemic cardiomyopathy | 11 | 90.9 | 63.6 | 27.3 | 18.2 |
| Liver non alcoholic steatohepatitis | 18 | 0.0 | 0.0 | 0.0 | 0.0 |
| Liver Steatohepatitis | 14 | 0.0 | 0.0 | 0.0 | 0.0 |
| Lung Idiopathic fibrosis | 17 | 100.0 | 100.0 | 100.0 | 100.0 |
| Lung-sarcoidosis | 21 | 4.8 | 0.0 | 0.0 | 0.0 |
| Muscle after immobilisation | 5 | 0.0 | 0.0 | 0.0 | 0.0 |
| Muscle from gastric cancer patient | 24 | 100.0 | 100.0 | 100.0 | 70.8 |
| Muscle in Sepsis | 13 | 0.0 | 0.0 | 0.0 | 0.0 |
| Ni allergy | 14 | 50.0 | 0.0 | 0.0 | 0.0 |
| Non-Diabetic heart failure | 12 | 0.0 | 0.0 | 0.0 | 0.0 |
| Normal Colon near tumour irradiated | 10 | 0.0 | 0.0 | 0.0 | 0.0 |
| Normal muscle Chronic Obstructive Pulmonary Disease patient | 30 | 83.3 | 53.3 | 16.7 | 0.0 |
| Normal Muscle Hormone replacement therapy | 10 | 0.0 | 0.0 | 0.0 | 0.0 |
| Normal muscle Metabolic syndrome | 12 | 0.0 | 0.0 | 0.0 | 0.0 |
| Normal Muscle obese | 23 | 56.5 | 30.4 | 0.0 | 0.0 |
| Normal Muscle Polycystic Ovary Syndrome | 26 | 38.5 | 11.5 | 0.0 | 0.0 |
| Normal Muscle post-gastric surgery | 3 | 66.7 | 66.7 | 33.3 | 33.3 |
| Normal Muscle pre-gastric surgery | 3 | 66.7 | 66.7 | 0.0 | 0.0 |
| Normal Muscle resveratrol | 10 | 0.0 | 0.0 | 0.0 | 0.0 |
| Pancreas Diabetes | 9 | 0.0 | 0.0 | 0.0 | 0.0 |
| Polymyositis | 5 | 20.0 | 0.0 | 0.0 | 0.0 |
| Psoriasis | 153 | 2.6 | 1.3 | 0.0 | 0.0 |
| Psoriasis treated | 44 | 0.0 | 0.0 | 0.0 | 0.0 |
| Pulmonary arterial hypertension with BMPR2 mutation | 2 | 0.0 | 0.0 | 0.0 | 0.0 |
| Skin Atopic dermatitis | 23 | 0.0 | 0.0 | 0.0 | 0.0 |
| Skin Atopic dermatitis Betamethasone | 10 | 0.0 | 0.0 | 0.0 | 0.0 |
| Skin Atopic dermatitis lesion | 9 | 0.0 | 0.0 | 0.0 | 0.0 |
| Skin Atopic dermatitis lesion UV light treated | 9 | 0.0 | 0.0 | 0.0 | 0.0 |
| Skin Atopic dermatitis Pimecrolimus | 10 | 0.0 | 0.0 | 0.0 | 0.0 |
| Skin alopecia areata | 5 | 0.0 | 0.0 | 0.0 | 0.0 |
| Skin Epidermolysis Bullosa | 6 | 0.0 | 0.0 | 0.0 | 0.0 |

*controls = healthy tissues taken from GeneAtlas U133A database
